# Supplementary material for: Navigating Nepal’s health financing system: A road to universal health coverage amid epidemiological and demographic transitions
Source: PLoS One. 2025 May 29;20(5):e0324880. doi: 10.1371/journal.pone.0324880 (PMC12121754; doi:10.1371/journal.pone.0324880)
Supplement: S2 Table — (DOC) [file pone.0324880.s002.doc]

**S2** **Table: Search strategy**

| Database | Keywords |
| --- | --- |
| PubMed | (("Social protection" OR "health financing"[Title/Abstract] OR "health insurance"[Title/Abstract]) AND ("Universal health coverage"[Title/Abstract] OR "Population coverage"[Title/Abstract] OR "service coverage"[Title/Abstract] OR "financial risk protection"[Title/Abstract] OR "financial health coverage"[Title/Abstract])) AND (Nepal*[Title/Abstract]) |
| Embase | ('social protection':ab,ti OR 'health financing':ab,ti OR 'health insurance':ab,ti) AND ('universal health coverage':ab,ti OR 'population coverage':ab,ti OR 'service coverage':ab,ti OR 'financial risk protection':ab,ti OR 'financial health coverage':ab,ti) AND 'nepal':ab,ti |
| Scopus | ( ABS ( "Social protection" OR "health financing" OR "health insurance" ) AND ABS ( "Universal health coverage" OR "Population coverage" OR "service coverage" OR "financial risk protection" OR "financial health coverage" ) AND ABS ( nepal* ) ) |
| Google scholar | “Social protection” OR “health financing” OR “health insurance”,  “Universal health coverage” OR “Population coverage” OR “service coverage” OR “financial risk protection” OR “financial health coverage”, Nepal* |
