# Supplementary material for: Navigating Nepal’s health financing system: A road to universal health coverage amid epidemiological and demographic transitions
Source: PLoS One. 2025 May 29;20(5):e0324880. doi: 10.1371/journal.pone.0324880 (PMC12121754; doi:10.1371/journal.pone.0324880)
Supplement: S3 Table — (DOCX) [file pone.0324880.s003.docx]

**S3 Table: Selected documents and reports in the review**

| **Author** | **Year** | **Title** | **Document type** |
| --- | --- | --- | --- |
| His Majesty's Government | 1990 | Constitution of the Kingdom of Nepal 1990 | Government Document |
| Ministry of Health and Population | 1991 | National Health Policy 1991 | Government Document |
| Ministry of Health and Population | 2005 | Nepal Health Sector Strategy, 2004-2009 | Government Document |
| Government of Nepal | 2007 | The Interim Constitution of Nepal 2007 | Government Document |
| Ministry of Health and Population | 2010 | Nepal Health Sector Strategy, 2010-2015 | Government Document |
| S. Witter, S. Khadka, H. Nath and S. Tiwari | 2011 | The national free delivery policy in Nepal: early evidence of its effects on health facilities | Journal Article |
| P. Gartoulla, T. Liabsuetrakul, V. Chongsuvivatwong and E. McNeil | 2012 | Ability to pay and impoverishment among women who give birth at a University Hospital in Kathmandu, Nepal | Journal Article |
| MOHP and NHSSP | 2012 | Review of national health policy 1991 | Report |
| M. Stoermer, F. Fuerst, K. Rijal, R. Bhandari, C. Nogier, G. S. Gautam, J. Hennig, J. Hada and S. Sharma | 2012 | Review of community-based health insurance initiatives in Nepal | Report |
| D. Daniels, K. Ghimire, P. Thapa, M. Réveillon, D. R. Pathak, K. Baral and N. Hamid | 2013 | Nepal Health Sector Programme II Mid-Term Review | Report |
| Ministry of Health and Population | 2013 | A review of studies on Nepal’s national free health care programme | Report |
| R. K. Dulal, A. Magar, S. D. Karki, D. Khatiwada and P. K. Hamal | 2014 | Analysis of Health Sector Budget of Nepal | Journal Article |
| Government of Nepal | 2014 | Budget Details: Red Book (Fiscal year 2010/11) | Government Document |
| I. Gupta and S. Chowdhury | 2014 | Correlates of out-of-pocket spending on health in Nepal: implications for policy | Journal Article |
| Ministry of Health and Population | 2014 | Progress Report on Financial Management In Nepal’s Health Sector | Report |
| Ministry of Health and Population | 2014 | National Health Policy 2014 | Government Document |
| Ministry of Health and Population | 2014 | National Health Insurance Policy 2014 | Government Document |
| G. o. Nepal | 2014 | Social Health Security Development Board Development Board (formation) Order 2014 | Government Document |
| E. Saito, S. Gilmour, M. M. Rahman, G. S. Gautam, P. K. Shrestha and K. Shibuya | 2014 | Catastrophic household expenditure on health in Nepal: a cross-sectional survey | Journal Article |
| Ministry of Health and Population | 2015 | Nepal Health Sector Strategy, 2015-2022 | Government Document |
| Ministry of Health and Population | 2015 | Partnership, Alignment and Harmonisation in the Health Sector | Report |
| S. R. Mishra, P. Khanal, D. K. Karki, P. Kallestrup and U. Enemark | 2015 | National health insurance policy in Nepal: challenges for implementation | Journal Article |
| Secretariat of Constituent Assembly | 2015 | Constitution of Nepal 2015 | Government Document |
| Ministry of Health and Population | 2016 | Working procedure related to citizen relief, compensation and financial support | Government Document |
| NHSSP | 2016 | Innovative Good Practices in Nepal’s Health Sector | Report |
| S. Uprety and B. Lamichhane | 2016 | Health budgeting and financing in Nepal: policy perspectives | Journal Article |
| T. Ensor, H. Bhatt and S. Tiwari | 2017 | Incentivizing universal safe delivery in Nepal: 10 years of experience | Journal Article |
| Government of Nepal | 2017 | Health Insurance Act | Government Document |
| C. L. Ranabhat, C. B. Kim, D. R. Singh and M. B. Park | 2017 | A Comparative Study on Outcome of Government and Co-Operative Community-Based Health Insurance in Nepal | Journal Article |
| M. Ghimire, R. Ayer and M. Kondo | 2018 | Cumulative incidence, distribution, and determinants of catastrophic health expenditure in Nepal: results from the living standards survey | Journal Article |
| Government of Nepal | 2018 | Public Health Service Act | Government Document |
| N. Kandel | 2018 | Nepal Health Insurance Bill: Possible Challenges and Way Forwards | Journal Article |
| Ministry of Health and Population | 2018 | Annual Health Report 2016/17 | Government Document |
| Ministry of Health and Population | 2018 | Nepal National Health Accounts 2015/16 report | Report |
| Ministry of Health and Population | 2018 | Nepal Health Sector Strategy (NHSS) Mid Term Review Report | Report |
| MoH and NHSSP | 2018 | Budget Analysis of Ministry of Health FY 2017/18 | Report |
| R. Pokharel and P. R. Silwal | 2018 | Social health insurance in Nepal: A health system departure toward the universal health coverage | Journal Article |
| P. Pyakurel, J. P. Tripathy, M. M. Oo, B. Acharya, U. Pyakurel, S. B. Singh, L. Subedi, K. P. Yadav, M. Poudel, D. R. Pandey, S. S. Budhathoki, G. R. Lohani and N. Jha | 2018 | Catastrophic health expenditure among industrial workers in a large-scale industry in Nepal, 2017: a cross-sectional study | Journal Article |
| K. T. Swe, M. M. Rahman, M. S. Rahman, E. Saito, S. K. Abe, S. Gilmour and K. Shibuya | 2018 | Cost and economic burden of illness over 15 years in Nepal: A comparative analysis | Journal Article |
| K. P. Acharya and R. T. Wilson | 2019 | Antimicrobial resistance in Nepal | Journal Article |
| S. Acharya, S. Ghimire, E. M. Jeffers and N. Shrestha | 2019 | Health Care Utilization and Health Care Expenditure of Nepali Older Adults | Journal Article |
| N. Adhikari, R. R. Wagle, D. R. Adhikari, P. Thapa and M. Adhikari | 2019 | Factors Affecting Enrolment in the Community Based Health Insurance Scheme of Chandranigahapur Hospital of Rautahat District | Journal Article |
| P. Ghimire, V. P. Sapkota and A. K. Poudyal | 2019 | Factors Associated with Enrolment of Households in Nepal's National Health Insurance Program | Journal Article |
| Government of Nepal | 2019 | Health Insurance Regulation | Government Document |
| G. N. Khanal | 2019 | Conditional cash transfer policies in maternal health service utilization in Nepal: Analysis of safe delivery incentive program (Aama Surakshya Karyakram) using Kingdon's multiple streams framework | Journal Article |
| P. Khanal and S. R. Mishra | 2019 | Federal governance and the undying parade for universal health coverage in Nepal | Journal Article |
| S. R. Khatiwoda, R. R. Dhungana, V. P. Sapkota and S. Singh | 2019 | Estimating the Direct Cost of Cancer in Nepal: A Cross-Sectional Study in a Tertiary Cancer Hospital | Journal Article |
| Ministry of Health and Population | 2019 | National Health Policy 2019 | Government Document |
| Ministry of Health and Population | 2019 | Strategic Review of Social Audit in the Health Sector | Report |
| Ministry of Health and Population | 2019 | Nepal National Health Accounts 2016/17. | Government Document |
| Ministry of Home Affairs | 2019 | Standards Related toTreatment Expenses, Relief, and Financial Assistance, 2077 | Government Document |
| W. B. MOHP, WHO, GIZ, | 2019 | Situation Analysis of Health Financing in Nepal | Report |
| D. R. Paudel | 2019 | Factors Affecting Enrollment in Government Health Insurance Program in Kailali District | Journal Article |
| C. L. Ranabhat, C. B. Kim, A. Singh, D. Acharya, K. Pathak, B. Sharma and S. R. Mishra | 2019 | Challenges and opportunities towards the road of universal health coverage (UHC) in Nepal: a systematic review | Journal Article |
| D. Acharya, B. Devkota, K. Gautam and R. Bhattarai | 2020 | Association of information, education, and communication with enrolment in health insurance: a case of Nepal | Journal Article |
| T. B. Adhikari, P. Acharya, M. Hogman, D. Neupane, A. Karki, A. Drews, B. G. Cooper, T. Sigsgaard and P. Kallestrup | 2020 | Prevalence of Chronic Obstructive Pulmonary Disease and its Associated Factors in Nepal: Findings from a Community-based Household Survey | Journal Article |
| Central Bureau of Statistics (CBS) | 2020 | Nepal Multiple Indicator Cluster Survey 2019,Survey Findings Report | Government Document |
| Employee Provident Fund | 2020 | Annual Report of Employee Provident Fund | Report |
| P. Gartaula, S. Neupane, D. N. Thakur and R. K. Sangroula | 2020 | Out of pocket Expenditure on Health Service Delivery at a Tertiary Care Women's Hospital: A Descriptive Cross-sectional Study | Journal Article |
| Government of Nepal | 2020 | Public Health Service Regulation 2020 (unofficial translation) | Government Document |
| B. Gyawali, P. Khanal, S. R. Mishra, E. van Teijlingen and D. Wolf Meyrowitsch | 2020 | Building Strong Primary Health Care to Tackle the Growing Burden of Non-Communicable Diseases in Nepal | Journal Article |
| Ministry of Health and Population | 2020 | Standard Treatment Protocol (STP) For Basic Health Services (BHS) Package 2078 | Government Document |
| Ministry of Health and Population | 2020 | Standard Treatment Protocol of Emergency Health Service Package | Government Document |
| C. L. Ranabhat, R. Subedi and S. Karn | 2020 | Status and determinants of enrollment and dropout of health insurance in Nepal: an explorative study | Journal Article |
| MoHP and UKaid/NHSSP | 2020 | Budget Analysis of Health Sector | Report |
| Ministry of Health and Population | 2020/21 | Annual Health Report 2020/21 | Government Document |
| A. Atreya, D. B. Shrestha, P. Budhathoki and S. Nepal | 2021 | Epidemiology of Road Traffic Accidents in Nepal from 2009/10 to 2019/20: A 10 Year Study | Journal Article |
| U. P. Bhusal and V. P. Sapkota | 2021 | Predictors of health insurance enrolment and wealth-related inequality in Nepal: evidence from Multiple Indicator Cluster Survey (MICS) 2019 | Journal Article |
| G. N. Khanal and R. B. Khatri | 2021 | Burden, prevention and control of tobacco consumption in Nepal: a narrative review of existing evidence | Journal Article |
| Ministry of Health and Population | 2021 | Directives, Guidelines and Manuals | Government Document |
| Nepal Health Research Council; Ministry of Health and Population, Institute for Health Metrics and Evaluation and Monitoring Evaluation and Operational Research | 2021 | Nepal Burden of Disease 2019: A Country Report based on the 2019 Global Burden of Disease Study | Report |
| S. Pandey and A. Daley | 2021 | Free delivery care and supply-side incentives in Nepal’s poorest districts: The effect on prenatal care and neonatal tetanus vaccinations | Journal Article |
| Reporter Swasthya Khabar Pratika | 2021 | Government's decision to bear former Prime Minister Khanal's medical expenses, violating the provisions of the Act | Newspaper Article |
| A. Shrestha, R. Maharjan, B. M. Karmacharya, S. Bajracharya, N. Jha, S. Shrestha, A. Aryal, P. P. Baral, R. D. Bhatt, S. Bhattarai, D. Bista, D. Citrin, M. Dhimal, A. L. Fitzpatrick, A. K. Jha, R. M. Karmacharya, S. Mali, T. Neupane, N. Oli, R. Pandit, S. B. Parajuli, P. M. S. Pradhan, D. Prajapati, M. Pyakurel, P. Pyakurel, B. K. Rai, B. P. Sapkota, S. Sapkota, A. Shrestha, A. P. Shrestha, R. Shrestha, G. N. Sharma, S. Sharma, D. Spiegelman, P. S. Suwal, B. Thapa, A. Vaidya, D. Xu, L. L. Yan and R. Koju | 2021 | Health system gaps in cardiovascular disease prevention and management in Nepal | Journal Article |
| A. Shrestha, S. B. Parajuli, A. Aryal, A. Shrestha, R. Maharjan, N. Jha, S. Bajracharya, S. Shrestha, T. Neupane, U. Poudel, B. M. Karmacharya, R. P. Koju and M. Dhimal | 2021 | National Needs Assessment of Health Information System to Address Cardiovascular Diseases in Nepal: A Mixed Method Study | Journal Article |
| A. K. Thapa and A. R. Pandey | 2021 | National and Provincial Estimates of Catastrophic Health Expenditure and its Determinants in Nepal | Journal Article |
| United Nations International Children's Emergency Fund | 2021 | Budget Brief Updates. Health Budget: FY 2021/22 | Report |
| World Health Organization | 2021 | Tracking Universal Health Coverage: global monitoring report | Report |
| D. Acharya, B. Devkota and G. L. Kreps | 2022 | Does perceived susceptibility and severity of health problems serve as drivers for household enrolment in health insurance? A case study from Nepal | Journal Article |
| B. Adhikari, S. R. Mishra and R. Schwarz | 2022 | Transforming Nepal's primary health care delivery system in global health era: addressing historical and current implementation challenges | Journal Article |
| H. Gardner, G. Miles, A. Saleem, A. Dunin-Borkowska, H. Mohammad, N. Puttick, S. Aksha, S. Bhattarai and C. Keene | 2022 | Social determinants of health and the double burden of disease in Nepal: a secondary analysis | Journal Article |
| G. B. Gurung and A. Panza | 2022 | Implementation bottlenecks of the National Health Insurance program in Nepal: Paving the path towards Universal Health Coverage: A qualitative study | Journal Article |
| G. B. Gurung and A. Panza | 2022 | Predictors of annual membership renewal to increase the sustainability of the Nepal National Health Insurance program: A cross-sectional survey | Journal Article |
| IQAir | 2022 | Air quality in Nepal Air quality in Nepal | Online Database |
| I. Karmacharya, S. Ghimire, K. Bhujel, A. Shrestha Dhauvadel, S. Adhikari, S. Baral and N. Shrestha | 2022 | Health Services Utilization among Older Adults in Pokhara Metropolitan City | Journal Article |
| Ministry of Health and Population | 2022 | Annual Health Report 2021/2022 | Government Document |
| Ministry of Health and Population | 2022 | Nepal Health Sector Strategic Plan, 2022-2031 | Government Document |
| Ministry of Health and Population | 2022 | Nepal Health Sector Support Programme 2017-2022 Achievements, Learning and Future Approaches: Results Brief | Report |
| Ministry of Health and Population | 2022 | Nepal National Health Accounts 2017/18 report | Report |
| Ministry of Health and Population | 2022 | Nepal National Health Accounts 2017/18. | Government Document |
| Ministry of Health and Population and Nepal Health Sector Support Programme | 2022 | Budget Analysis of Ministry of Health FY 2021/22 | Report |
| Ministry of Health and Population and NHSSP | 2022 | Health Sector Budget Analysis: First Five Years of Federalism | Government Document |
| Nepal Health Research Council | 2022 | Towards Universal Health Coverage: Addressing Financial Hardship and Improv ing Access to Healthcare in Nepal (Policy brief) | Report |
| NHRC | 2022 | Towards Universal Health Coverage: Addressing Financial Hardship and Improving Access to Healthcare in Nepal (Policy brief) | Report |
| S. Rai, S. Gautam, G. K. Yadav, S. R. Niraula, S. B. Singh, R. Rai, S. Poudel and R. B. Sah | 2022 | Catastrophic health expenditure on chronic non-communicable diseases among elder population: A cross-sectional study from a sub-metropolitan city of Eastern Nepal | Journal Article |
| K. Rana, R. Chimoriya, N. B. Haque, M. K. Piya, R. Chimoriya, M. Ekholuenetale and A. Arora | 2022 | Prevalence and Correlates of Underweight among Women of Reproductive Age in Nepal: A Cross-Sectional Study | Journal Article |
| B. P. Sapkota, K. P. Baral, U. Berger, K. G. Parhofer and E. A. Rehfuess | 2022 | Health sector readiness for the prevention and control of non-communicable diseases: A multi-method qualitative assessment in Nepal | Journal Article |
| S. Shah, N. Jha, V. K. Khanal, G. Nepal Gurung, B. Sharma and M. Shrestha | 2022 | Utilization of social health security scheme among the households of Illam district, Nepal | Journal Article |
| P. Sharma, D. K. Yadav, N. Shrestha and P. Ghimire | 2022 | Dropout Analysis of a National Social Health Insurance Program at Pokhara Metropolitan City, Kaski, Nepal | Journal Article |
| WHO South-East Asia Region | 2022 | Monitoring progress on universal health coverage and the health-related Sustainable Development Goals in the WHO South-East Asia Region: 2022 update | Report |
| B. Adhikari, A. R. Pandey, B. Lamichhane, S. P. KC, D. Joshi, S. Regmi, S. Giri and S. C. Baral | 2023 | Non-Communicable Disease Service Readiness in Nepal: A Further Analysis of Nepal Health Facility Survey-2021 | Journal Article |
| K. K. Aryal | 2023 | Free Basic Healthcare in Nepal: Can the Dream be Turned into Reality in the Pursuit of Universal Health Coverage? | Journal Article |
| M. B. Basnet | 2023 | Access to the poor's money, who took how much (list) (Nepali) | Newspaper Article |
| S. Ghimire, S. Ghimire, P. Khanal, R. A. Sagtani and S. Paudel | 2023 | Factors affecting health insurance utilization among insured population: evidence from health insurance program of Bhaktapur district of Nepal | Journal Article |
| Gyanwali S | 2023 | Status, challenges and way forward for Health Insurance Programme in Nepal (Inv Talk) | Conference Paper |
| Institute for Health Metrics and Evaluation | 2023 | Nepal, All-cause, Spending as % of GDP, 1995-2024 | Online Database |
| G. N. Khanal, B. Bharadwaj, N. Upadhyay, T. Bhattarai, M. Dahal and R. B. Khatri | 2023 | Evaluation of the National Health Insurance Program of Nepal: are political promises translated into actions? | Journal Article |
| Ministry of Health and Population | 2023 | National Health Financing Strategy, 2023-2033 | Government Document |
| Ministry of Health and Population | 2023 | Nepal National Health Accounts 2018/19 – 2019/20 | Government Document |
| Ministry of Health and Population, New ERA and ICF | 2023 | 2022 Nepal Demographic and Health Survey Key Findings | Report |
| Ministry of Health and Population, NHSSP and Options | 2023 | Nepal Health Sector Support Programme (NHSSP 3) – No Cost Extension | Report |
| National Centre for AIDS and STD Control | 2023 | National HIV Factsheet | Report |
| Nepal Health Research Council | 2023 | Evidence on Social Health Protectionfor strong institutions (Panel discussion) | Conference Paper |
| Our World in Data | 2023 | Country profile:Nepal | Online Database |
| A. Pandey , P. Baral and A. Shrestha | 2023 | Non-Communicable Diseases and their risk factors: panel discussion on its interventions in Nepal | Conference Paper |
| A. R. Pandey | 2023 | Health financing reforms in the quest for universal health coverage: Challenges and opportunities in the context of Nepal | Journal Article |
| A. Paudel | 2023 | Work on 400 basic hospitals in local units may not start next fiscal year | Newspaper Article |
| V. P. Sapkota, U. P. Bhusal and G. P. Adhikari | 2023 | Occupational and geographical differentials in financial protection against healthcare out-of-pocket payments in Nepal: Evidence for universal health coverage | Journal Article |
| Social Security Fund | 2023 | Daily summary report as of 7 October 2023 | Report |
| S. P. Wasti, E. van Teijlingen, S. Rushton, M. Subedi, P. Simkhada, J. Balen and T. Nepal Federal Health System | 2023 | Overcoming the challenges facing Nepal's health system during federalisation: an analysis of health system building blocks | Journal Article |
| World Health Organization | 2023 | Tracking universal health coverage: 2023 global monitoring report | Report |
| International Labour Organization | 2023 | Social health protection in Nepal. State of play and recommendations towards universal extension of coverage | Report |
| D. Acharya, S. Sharma and K. Bietsch | 2024 | Enrollment and associated factors of the national health insurance program of Nepal: Further analysis of the Nepal Demographic and Health Survey 2022 | Journal Article |
| R. Adhikari, R. Shah and V. Khanal | 2024 | Older people's challenges accessing health services in central Nepal: a qualitative study using the domain of access framework | Journal Article |
| R. Ayer, S. Tiwari, S. P. Jnawali and R. Dael | 2024 | A Study on Nepal’s National Health Insurance Program | Report |
| Employee Provident Fund | 2024 | Annual Report of Employee Provident Fund 2022/2023 | Report |
| EPF | 2024 | Healthcare Plan | Report |
| S. Ghimire, S. Ghimire, D. R. Singh, R. A. Sagtani and S. Paudel | 2024 | Factors influencing the utilisation of National health insurance program in urban areas of Nepal: Insights from qualitative study | Journal Article |
| Health Insurance Board | 2024 | Acts, rules, directives and procedures | Government Document |
| Ministry of Health and Population | 2024 | Progress of health and population sector 2023/24 | Government Document |
| Ministry of Health and Population | 2024 | National joint annual review report | Government Document |
| Ministry of Health and Population | 2024 | Annual Health Report 2022/2023 | Report |
| Ministry of Health and Population | 2024 | Geriatrics (Senior Citizens) Health Service Operational Guideline- 2023/24 | Government Document |
| National Statistics Office | 2024 | Nepal Living Standard Survey IV 2022/23 | Report |
| P. Poudel, R. Khatri, L. Bhatt, P. Thapa, R. K. Mishra, S. Tuladhar and E. Panahi | 2024 | Baseline Status of Basic Health Service Delivery, 2022 Nepal DHS and 2021 Nepal HFS. DHS Further Analysis Reports No. 157 | Report |
| R. Shrestha, A. Shakya, P. Khanal, V. K. Khanal, N. Jha, G. Nepal Gurung and L. Subedi | 2024 | User satisfaction with the National Health Insurance Program: A community-based survey from the Ilam district of Nepal | Journal Article |
| Social Security Fund | 2024 | Daily Summary Report as of 09 Jan 2024 | Government Document |
| Health Insurance Board | 2024 | Annual report 2022/23: factsheet and executive summary | Government Document |
| Health Insurance Board | 2024 | Operational guideline for implementation of co-payment system in the health insurance program, 2080 | Government Document |
| Health Insurance Board | 2024 | Accreditation guideline for service providing facilities, 2024 | Government Document |
| Ministry of Health and Population | 2024 | Annual report 2022/23: factsheet and executive summary | Government Document |
| World Health Organization | 2024 | The Global Health Observatory: Explore a world of health data | Online Database |
| Ministry of Health and Population | 2025 | Medical treatment of deprived citizens | Web Page |
| Social Security Fund | 2024 | Annual Report 2023/2024 | Government Document |
| Ministry of Health and Population | 2021 | Senior Citizen Health Service Operation Guideline 2077 (2021) | Government Document |
| Ministry of Health and Population | 2020 | Social Service Unit Establishment and Operational Guidelines | Government Document |
| Health Insurance Reform Proposal Task Force | 2025 | Health Insurance Reform Proposal Task Force Report, 2081 (2025), Nepali version | Goverment Document |
