# Supplementary figures and images for: Navigating Nepal’s health financing system: A road to universal health coverage amid epidemiological and demographic transitions
Source: PLoS One. 2025 May 29;20(5):e0324880. doi: 10.1371/journal.pone.0324880 (PMC12121754; doi:10.1371/journal.pone.0324880)

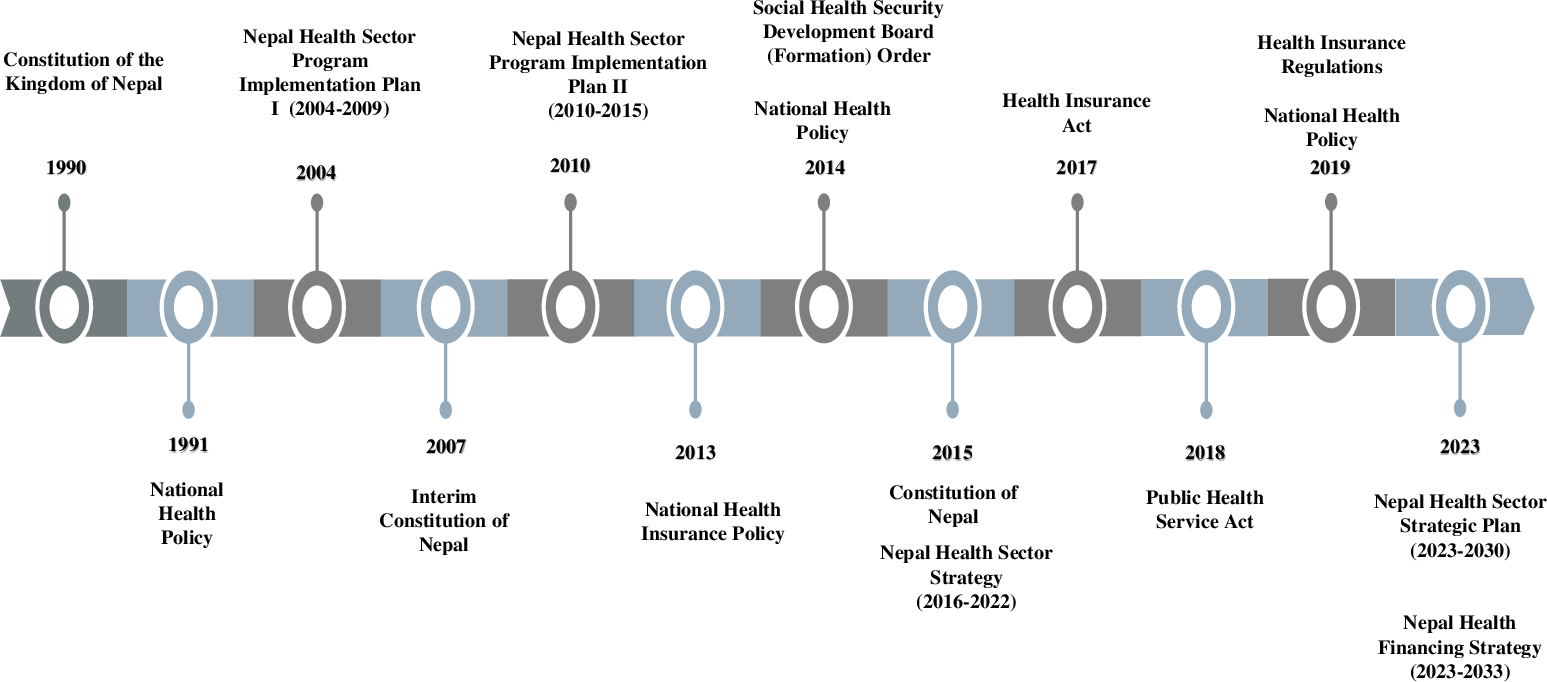

Supplement: S1 Fig — (TIF) [file pone.0324880.s004.tif]

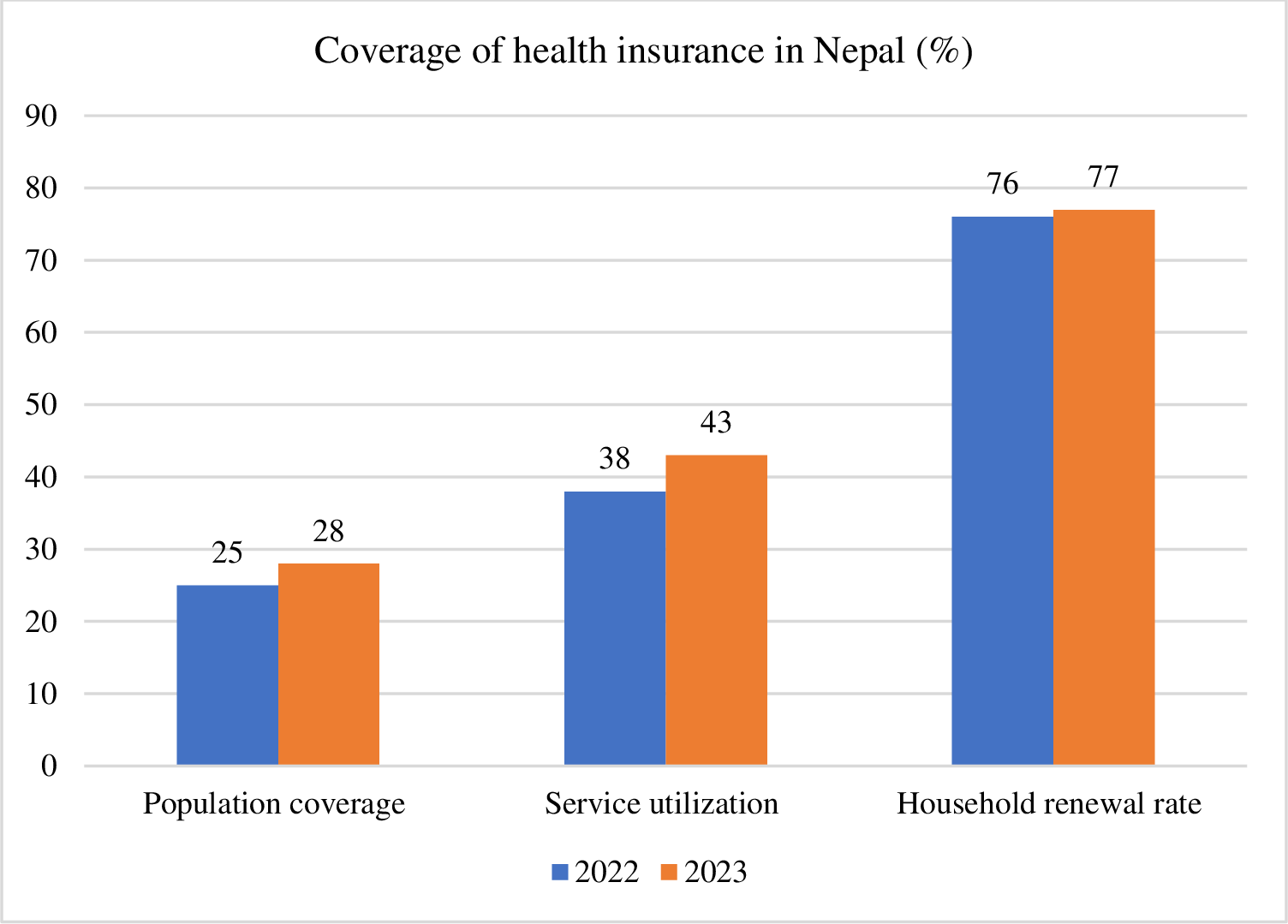

Supplement: S2 Fig — (TIF) [file pone.0324880.s005.tif]

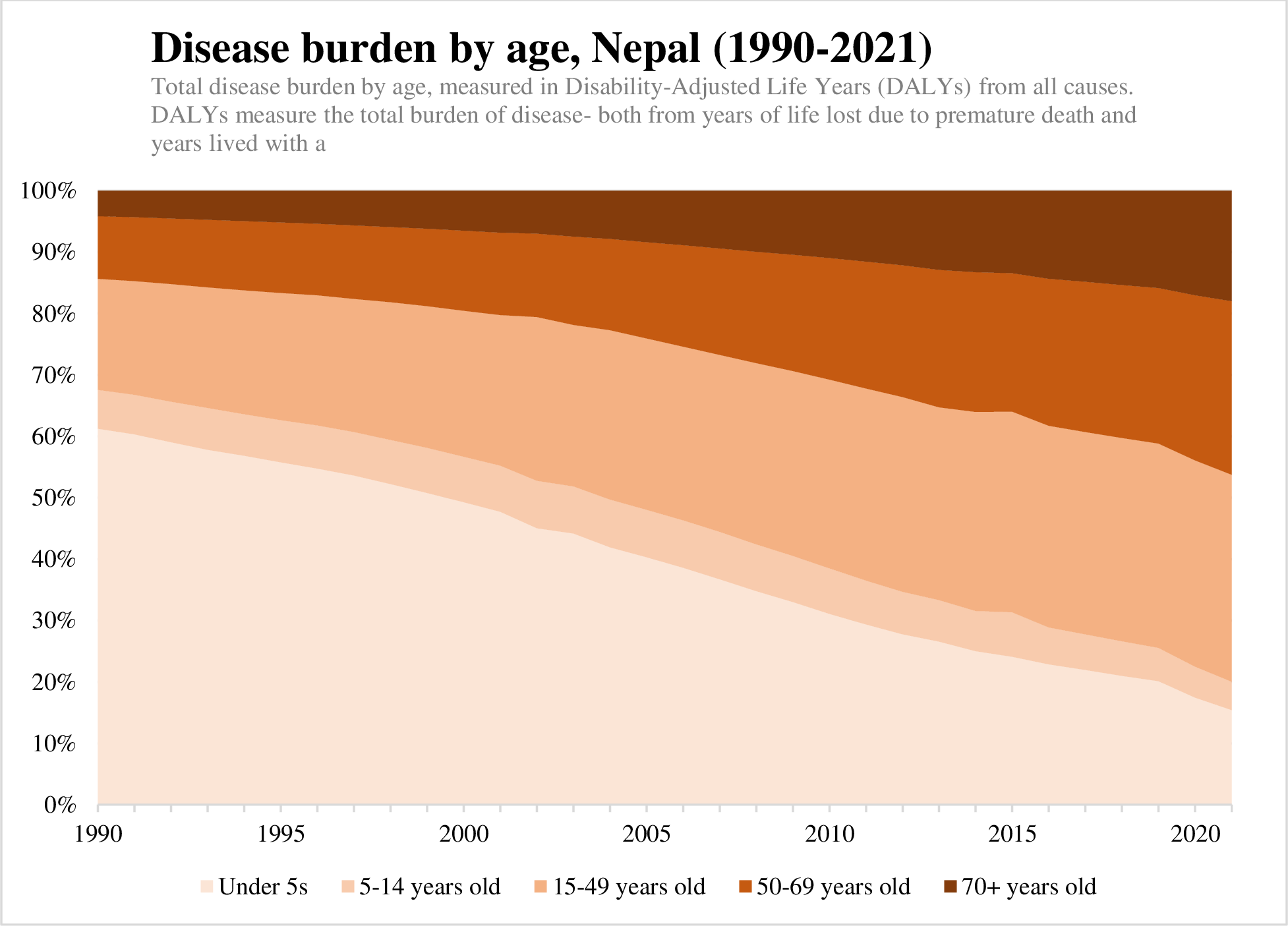

Supplement: S3 Fig — (TIF) [file pone.0324880.s006.tif]

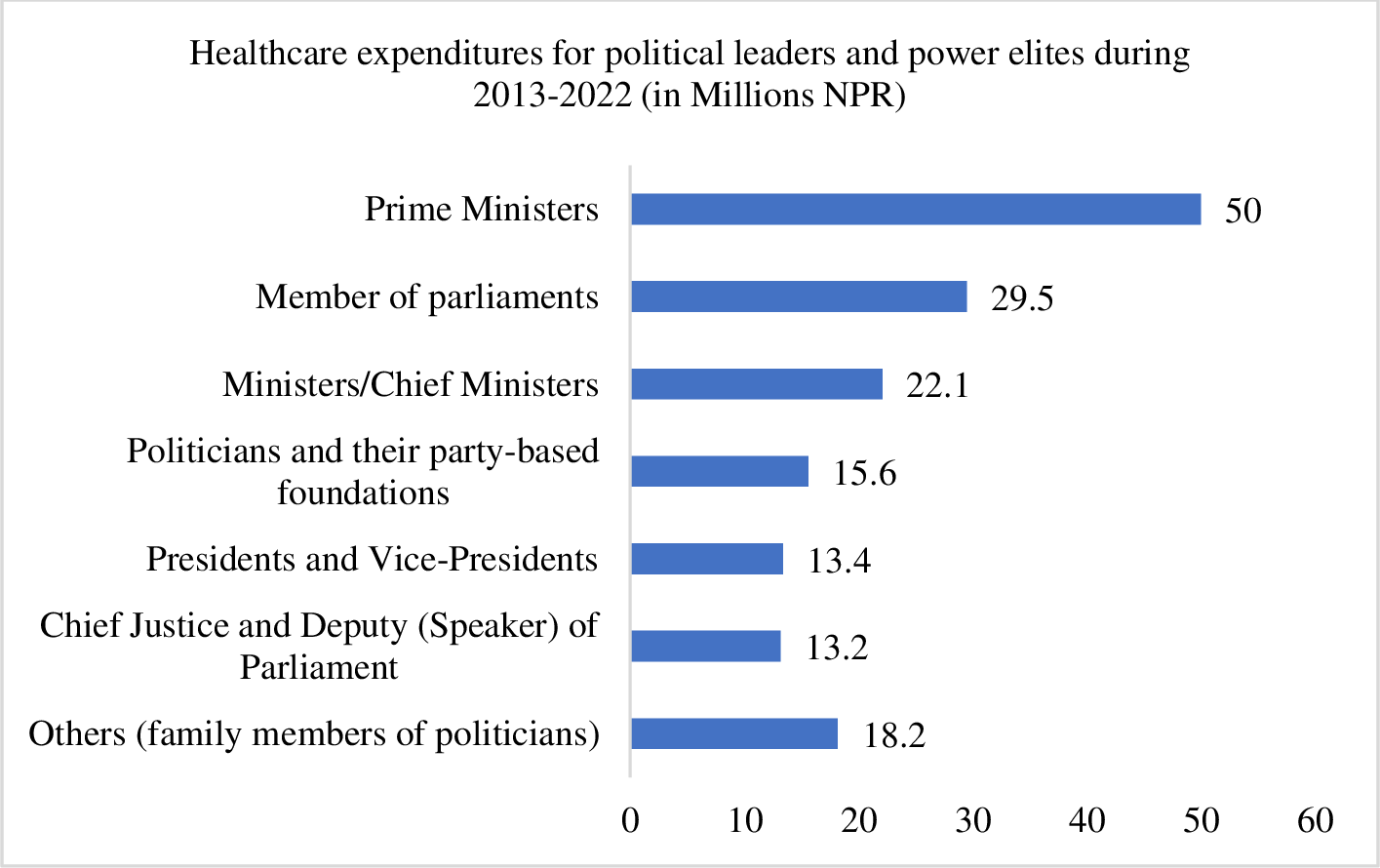

Supplement: S4 Fig — (TIF) [file pone.0324880.s007.tif]
