# Supplementary material for: Navigating Nepal’s health financing system: A road to universal health coverage amid epidemiological and demographic transitions
Source: PLoS One. 2025 May 29;20(5):e0324880. doi: 10.1371/journal.pone.0324880 (PMC12121754; doi:10.1371/journal.pone.0324880)
Supplement: Attachment — Submitted filename: Point by point response_R2_rev.pdf [file pone.0324880.s011.pdf]

Point by point response to reviewer comments

Submission ID: [PONE-D-24-23054R1] - [EMID:dd6ac99af9acd14a]

Title: Navigating Nepal's Health Financing System: A Path to Universal Health Coverage Amid Epidemiological and Demographic Transitions

Dear Editor and Reviewers,

We extend our heartfelt gratitude to you for diligently managing and evaluating our manuscript. Your insightful comments and valuable recommendations have significantly enhanced the quality of our work. In response to the reviewers' feedback, we have meticulously revised the manuscript, addressed each comment and made the necessary modifications.

Additionally, we have attached a comprehensive response letter that outlines how we have incorporated the reviewers' suggestions into the revised manuscript. **Authors' responses are italicized text for clarity.**

Thank you once again for your time and effort in improving our manuscript.

……………………………………..

Sincerely,

Resham B. Khatri, PhD MPH MA

on behalf of all co-authors

Reviewer #1: The authors have addressed all my comments well. I have no further comments. Good job. Looking forward to reading the published version if accepted.

> *Author's response: authors team would like to thank reviewer for this feedback, thank you so much.*

Reviewer #2: Thank you for your revisions. The manuscript has improved in structure and clarity, but a few areas still need refinement to strengthen its academic rigor and policy relevance. Below are specific suggestions for improvement:

Author's response: The authors team would like to thank reviewer for this feedback. Authors would like to appreciate reviewer for this opportunity to revise further our manuscript.

1. Introduction
The introduction provides useful background, but the research gap is not clearly stated. Consider adding a direct statement on what knowledge gap this study fills, particularly how it builds on or differs from existing health financing research in Nepal.

Author's response: we appreciate and thank reviewer for this comment. We further trimmed and focus the of this research. We have revised the introduction adding a short paragraph on knowledge gaps and significance of this study at end of the introduction section.

2. Methods
The PRISMA-ScR framework is well presented, but there is no mention of how data discrepancies were handled during extraction. Please include a short statement on how inconsistencies in data collection and classification were resolved to improve transparency and reproducibility.

Author's response: We again thank reviewer for this feedback. We have added statement and described how data discrepancy was handled during extraction and analysis process. Refer to the "Extraction and charting of data" section in page 8.

3. Results
Some sections of the results are too narrative-heavy despite already having well-presented figures and tables. Reduce excessive text where data is already visualized to improve readability.

Author's response: In agreement with the reviewer's suggestions, we have significantly revised the results section to minimize the text that was already presented in tables and figures. This results table provides an opportunity for a robust discussion on how health financing functions should be structured in terms of risk pooling, payment mechanisms, and strategic purchasing.

4. Discussion
The discussion remains descriptive rather than analytical. Strengthen this section by discussing why certain health financing interventions have/have not worked in Nepal, linking findings to structural or policy constraints. A comparative benchmark would add value—how does Nepal's health financing situation compare with similar LMICs in South Asia? (This was A more critical evaluation of government policies and health financing gaps is needed to make the discussion more insightful.

Author's response: We greatly appreciate this feedback. Thank you, Reviewer. We have revised the discussion section to include some critique on the overall policy framing of

Nepal's health financing system (in line with constitutional, policy, and strategic development). This is followed by an analysis of existing health financing model (tax funded and premium based models), and then an analysis of policies and programs with respect to universal health coverage, strategies to strengthen the National Health Insurance Program (NHIP), and strategies to improve health financing functions in relation to revenue generation/ risk pooling, purchasing of services and payment mechanisms/resource allocation. We have also drawn lessons from health financing schemes in other countries with health system designs like Nepal. We believe the revised discussion section is more streamlined with the findings and aligns convincingly with the research question under inquiry.

5. Conclusion & Policy Recommendations- The conclusion is still too general and does not clearly outline next steps. Please add 2-3 specific policy takeaways, such as recommended financing mechanisms, potential regulatory adjustments, or areas needing further research. Policymakers should be able to extract actionable insights directly from this section.

> *Author's response: thank you reviewer, we have revised final conclusions and policy implications as suggested.*

6. Figures & Exhibits
Some figures and exhibits do not add analytical value and could be moved to supplementary materials or removed. Specifically, Figures 2, 4, and 6 can be safely removed or moved to supplementary material as they are redundant and do not directly support key arguments in the discussion.

> *Author's response: We have moved figures 2,4, and 6 as supplementary file.*

Reviewer #3: I have no further comments for the authors. I believe, this manuscript is now ready for publication.

> *Authors response. Author's response: authors team would like to thank reviewer for this feedback, thank you so much.*

Journal Requirements:

Please review your reference list to ensure that it is complete and correct. If you have cited papers that have been retracted, please include the rationale for doing so in the manuscript text or remove these references and replace them with relevant current references. Any changes to the reference list should be mentioned in the rebuttal letter that accompanies your revised manuscript. If you need to cite a retracted article, indicate the article's retracted status in the References list and include a citation and full reference for the retraction notice.

Author's response: authors team would like to thank editor for this feedback, we have rechecked references list again and ensure the journal requirement.
