# Supplementary material for: Navigating Nepal’s health financing system: A road to universal health coverage amid epidemiological and demographic transitions
Source: PLoS One. 2025 May 29;20(5):e0324880. doi: 10.1371/journal.pone.0324880 (PMC12121754; doi:10.1371/journal.pone.0324880)
Supplement: Attachment — Submitted filename: Point by point response_R3_rev.pdf [file pone.0324880.s012.pdf]

Point by point response to reviewer comments

Submission ID: [PONE-D-24-23054R2] - [EMID:dd6ac99af9acd14a]

Title: Navigating Nepal's Health Financing System: A Path to Universal Health Coverage Amid Epidemiological and Demographic Transitions

Dear Editor and Reviewer,

We would like to thank you for reviewing our manuscript and providing us important feedback that has helped to improve the quality of our work. In this revised submission, we have tried our best to address the reviewer's suggestions.

Additionally, we have attached a comprehensive response letter that outlines how we have incorporated the reviewers' suggestions into the revised manuscript.

Thank you once again for your time and effort in providing constructive feedback on our manuscript.

…………………………………..

Sincerely,

Resham B. Khatri, PhD MPH MA

on behalf of all co-authors

Reviewer #2: Thank you for incorporating the revisions. The manuscript has improved significantly in structure and clarity. Below are a few minor refinements that would further enhance the paper's academic rigor and impact:

1. Abstract

The abstract is now more structured, but it still lacks specific quantitative insights (e.g., exact figures on health financing gaps, enrollment rates). Consider incorporating key data points to strengthen the findings. The contribution of the study could be highlighted more explicitly to make it clearer why this study is valuable.

Thank you for highlighting the need to incorporate key data points in the findings section of the abstract. The updated abstract has now data points related to health financing situation of Nepal including out-of-pocket expenditure. Furthermore, the conclusion section in the abstract is refined to align with the changes made in the conclusion section of this paper.

2. Introduction
The introduction is better streamlined, but the research gap statement could be made clearer. A direct sentence explaining what this study adds beyond existing literature would improve clarity.
We now have improved this section by clearly spelling out the research gap and have highlighted the contribution of the study to the existing literature in the last paragraph of the introduction section

3. Methods
The PRISMA-ScR framework is well articulated, and the addition of a statement on data discrepancies is useful.
A brief mention of how grey literature bias was managed would further enhance the methodology section.
We tried to address data discrepancies by including figures from relevant authorities and incorporating latest figures. We also validated data and grey literature information from people who are closely acquainted with Nepal's health financing system. A short narrative of how grey literature bias was managed has been incorporated in the "Selection of documents" sub-heading under the "Methods" section.

4. Results
The results section is much improved, with better clarity and reduced redundancy. However, comparative benchmarks with other LMICs remain somewhat limited. Consider adding a brief comparison to place Nepal's health financing in a broader global context.. We appreciate the reviewer's suggestion to include comparative benchmarks with other LMICs in the results section. However, the authors believe that instead of highlighting these comparisons in the results, which primarily focus on Nepal's health financing context, we have incorporated them in the introduction. In the introduction, we provide a brief comparison of Nepal's health financing position within the broader global context, contrasting it with global and regional averages, as well as with data from other LMICs.

5. Discussion
The discussion still leans towards descriptive rather than analytical in some areas.
A stronger evaluation of why certain interventions have or have not worked in Nepal would add more depth.

The comparisons with other LMICs have improved, but linking them more explicitly to Nepal's policy context would further strengthen the analysis.
Thank you for your feedback. We have refined the discussion section by reorganizing the subheadings and removing redundant or descriptive text. To further strengthen the argument and provide context and recommendations for Nepal's health financing, we have included additional lessons and examples from other LMICs. Additionally, based on the findings and discussion of the study, we summarized and visually presented the implications for policy,

program, and research in a figure. We believe these revisions have enhanced the discussion, as suggested.

6. Conclusion & Policy Recommendations
The conclusion remains somewhat general and could be more action-oriented.
Please consider explicitly outlining 2-3 key policy recommendations that emerge from the study.
A short mention of next steps (e.g., future research areas, practical policy implementations) would add value.
In response to the reviewer's suggestions, we have reframed the conclusion to clearly summarize the key findings. Additionally, we have outlined four action-oriented policy recommendations aimed at addressing the existing gaps in health financing performance. We have also highlighted potential areas for future research that may be of interest to research stakeholders.
7. Figures & Exhibits
Figures 2, 4, and 6 have been moved to supplementary materials as suggested—this is a positive change.
Figure 10 is still included, but its necessity should be reconsidered. If it does not provide critical analysis, it might be more suitable for supplementary materials.
We have well-noted the feedback and have moved the figure 7 (figure 10 in previous track change version) in the supplementary section.

Final Suggestion
With these minor refinements, the manuscript will be well-positioned for acceptance. These improvements will ensure that the study contributes meaningful insights to the discussion on health financing in Nepal. Let me know if further clarifications are needed.
Thank you for this observation. We have tried our best to incorporate the feedback and we are hopeful that the study if published would be helpful to initiate discussion on health financing reforms in Nepal.
