# Supplementary material for: Navigating Nepal’s health financing system: A road to universal health coverage amid epidemiological and demographic transitions
Source: PLoS One. 2025 May 29;20(5):e0324880. doi: 10.1371/journal.pone.0324880 (PMC12121754; doi:10.1371/journal.pone.0324880)
Supplement: Attachment — Submitted filename: Plos one Sept 03092024.docx [file pone.0324880.s008.docx]

Reviewer report

**Title:** Financing of health policies and programs amid demographic and epidemiological

transitions in Nepal: a scoping review and synthesis of evidence using universal health

coverage framework

Main text

**Introduction**

1. The health system of Nepal was well described in the introduction. However, the introduction lacks a description of how the epidemiology has changed in the country. Could you provide details of the changes in the disease burden the country has experienced? The 2019 burden of disease report may be helpful. <https://nhrc.gov.np/wp-content/uploads/2022/02/BoD-Report-Book-includ-Cover-mail-6_compressed.pdf>
2. Page 5, Lines 100-101 “Nepal is afflicted by a double burden of both high infectious and non-communicable diseases (NCDs).” This statement is a contradiction of what you stated in the abstract (transition to NCDs etc but not dual burden). Clarify, for consistency.
3. The last two paragraphs in the introduction seem disjointed yet they are related. Including information on how the 2015 constitution of Nepal reflected the UHC ambitions and whether there are aspects of UHC that are not addressed by the constitution is likely to be beneficial to the reader.
4. The research gap that the study sought to address is not well elaborated in the introduction. You state that “Nonetheless, there is limited discussion about how health financing policies and strategies are in place to address these issues.” How limited is this discussion? Has there been any literature about the same? What financing policies are these, anyway? Have some studies reviewed some of these policies ready? And if yes, what did they find or what was the scope of the review? How different are they from this study? You should consider addressing these questions to give the reader a clear and well elaborated picture if the existing research gap that you are trying to fill with this study.
5. In the last two sentences of the introduction, you mention what you have done in this study and the likely implication. Rephrase the sentences and clearly state the aim of your study reflecting the scope (the status and challenges of financing of health policies and programs towards universal health coverage).
6. Ensure that the aim in the introduction and abstract are well aligned/the same.

**Methods**

7. The methods section is overly summarized which may have resulted into omission of relevant information for example in the search strategy: how were the search terms identified, were the search strings customised to the databases, was the search automated? What platforms were used if any? Concerning the selection of the articles: who did the selection, was it done by one or more people and how? What was the process for selecting the articles, was any platform used? Etc. Data extraction: how was the data extracted? Was it automated or done by individuals, did they use a template or program? For reproducibility, a more detailed methods section should be provided.
8. The study states that PRISMA-ScR guidelines were followed. It is great that the checklist was attached. The structure of the methods section should be aligned with the requirements of PRISMA-ScR guideline. This will also enable you have a more detailed methods section making it easy to follow. This could be done by including subheadings reflecting the main components of the guideline.
9. Specify the reference for the WHO UHC framework used as there are a number of variations for example a recent one <http://www.uhcwpr.info/>. Please include a website as part of the reference. Page 6, lines 132-136
10. Replace “and” with “or” in the statement “… in Nepali and English …” Page 6, line 137
11. Clearly state the key words used in the search. Were they “Health financing”, Universal Health Coverage” and “Nepal”?
12. State the date when the search was date.
13. Include details of the strategy used to search for grey literature both online and offline (if done).
14. Attach as supplementary material, the search strings used in searching the databases or platforms used.

**Results**

15. Include a table of characteristics of the included articles, and records
16. You applied the WHO UHC framework (UHC cube), I would suggest that you reflect this in table 1 too by splitting both the status and Issues and challenges columns into three columns each to reflect the three dimensions of the UHC framework.
17. Table 1: Some of the statements are general and could benefit from making them more specific. For example, when you state inequity, what aspect are you talking about, who are the disadvantaged, is it the poor, is it women? etc. When you mention poor public satisfaction with CBHI, what aspect of CBHI were people unhappy with, was it the service package, or something else? It will be helpful to be more specific in the Issues and challenges column.
18. Table 1: I would have expected you to include the National Health Insurance Act 2017 as one of the major health financing policies on the list in table 1. Was there a reason of leaving it out?
19. Table 2: Include a specific column for the name of the program that you are referring to in each row, separate from the actor(s) managing the program. This will make the table clearer and easier to follow.
20. Table 2: The scope of your study did not include functions but programs and policies. Please clarify why you are including health financing functions in the table.
21. Some of the sections provide explanation of the results. The explanation should be moved to the discussion. Page 20, Lines 160 to 171. Please cross check pages 15 to 20 to exclude the possible explanations provided and move them to the discussion section.
22. The results section seems to have references that were not part of the articles included in the study for example reference number 97 (IQAir. Air quality in Nepal Air quality in Nepal 2022). Such references and the respective statements they support should be moved and considered for the discussion section.

**Discussion**

23. The discussion explains the results to a great extent but misses comparing the findings in this study with similar studies that were done in Nepal or comparable settings. It will be great to include this comparison in the discussion.
24. One of the intentions of your study is to see whether there has been a mismatch between the health financing policies and the shifting demographics and disease burden in Nepal. However, this has not been discussed much and neither commented about in the first paragraph of discussion. Consider including a sub section on whether the policies are responding to the demographic and epidemiological shift and add a comment about the same in the first paragraph of the discussion section.
25. The sub section entitled “Policy and plans versus translation into programs and services” has content that does not fully reflect the title. The current content has elements beyond the scope of the title (page 22, lines 207-221) and omits other relevant content, it would be great to provide more relevant examples. Some of the content especially regarding the details on financial protection my fit better in the subsections that follow.
26. Some of the subsections should be considered for merging as they discuss a similar area for example “Health insurance program in limbo”, and “High OOP and catastrophic expenditure”.

**Strength and limitations**

27. Not interviewing people is part of the design of a scoping review in most cases so there is no need to mention it.
